# Selective Exposure of the Fetal Lung and Skin/Amnion (but Not Gastro-Intestinal Tract) to LPS Elicits Acute Systemic Inflammation in Fetal Sheep

**DOI:** 10.1371/journal.pone.0063355

**Published:** 2013-05-17

**Authors:** Matthew W. Kemp, Paranthaman Senthamarai Kannan, Masatoshi Saito, John P. Newnham, Tom Cox, Alan H. Jobe, Boris W. Kramer, Suhas G. Kallapur

**Affiliations:** 1 School of Women’s and Infants’ Health, The University of Western Australia, Perth, Australia; 2 Division of Pulmonary Biology, Cincinnati Children’s Hospital Medical Centre, University of Cincinnati School of Medicine, Cincinnati, Ohio, United States of America; 3 Division of Perinatal Medicine, Tohoku University Hospital, Sendai, Japan; 4 Department of Paediatrics, School of Oncology and Developmental Biology, Maastricht University Medical Centre, Maastricht, The Netherlands; Université de Montréal, Canada

## Abstract

Inflammation of the uterine environment (commonly as a result of microbial colonisation of the fetal membranes, amniotic fluid and fetus) is strongly associated with preterm labour and birth. Both preterm birth and fetal inflammation are independently associated with elevated risks of subsequent short- and long-term respiratory, gastro-intestinal and neurological complications. Despite numerous clinical and experimental studies to investigate localised and systemic fetal inflammation following exposure to microbial agonists, there is minimal data to describe which fetal organ(s) drive systemic fetal inflammation. We used lipopolysaccharide (LPS) from *E.coli* in an instrumented ovine model of fetal inflammation and conducted a series of experiments to assess the systemic pro-inflammatory capacity of the three major fetal surfaces exposed to inflammatory mediators in pregnancy (the lung, gastro-intestinal tract and skin/amnion). Exposure of the fetal lung and fetal skin/amnion (but not gastro-intestinal tract) caused a significant acute systemic inflammatory response characterised by altered leucocytosis, neutrophilia, elevated plasma MCP-1 levels and inflammation of the fetal liver and spleen. These novel findings reveal differential fetal organ responses to pro-inflammatory stimulation and shed light on the pathogenesis of fetal systemic inflammation after exposure to chorioamnionitis.

## Introduction

Preterm birth is a leading cause of perinatal morbidity and death in industrialised and developing nations alike, accounting for approximately 11% of births world-wide [1]. Globally, preterm birth accounts for 28% of the estimated 4 million neonatal deaths each year [2]. Both clinical and experimental studies strongly demonstrate a causal link between intrauterine infection (chorioamnionitis) and preterm labour [3]–[6].

Preterm infants are at an increased risk for several debilitating illnesses including chronic lung disease, retinopathy of prematurity, necrotizing enterocolitis, cerebral palsy and decreased IQ [5]–[13]. A major contributory factor for these neonatal morbidities is fetal inflammation, called the fetal inflammatory response syndrome (FIRS), which is characterised in clinical research by cord blood IL-6 in excess of 11 pg/mL [14], [15]. In contrast to chorioamnionitis alone, FIRS is indicative of a fetal response to infection and adverse neonatal outcomes [15].

In fetal sheep, an intraamniotic injection of LPS causes chorioamnionitis which also increases surfactant production and lung compliance [16]. In this model, preterm fetuses exposed to LPS have lung injury and remodelling [17], [18], brain injury [19], gastrointestinal injury [20] and skin inflammation [21] mimicking the human disease. Despite inflammatory process in internal organs, LPS was not detected in the blood of fetuses exposed to intraamniotic LPS [22], and an intravascular injection of LPS at a 10,000 fold lower dose compared to amniotic dose causes fetal death. These results imply that the LPS in the amniotic fluid transduces inflammation to internal fetal organs via the epithelial surfaces that are in contact with the amniotic fluid (lung, gastrointestinal tract, amniotic membrane, skin). Using surgery to isolate the organ response to LPS, we previously demonstrated that maturation of the fetal lung (as demonstrated by increases in lung surfactant and compliance) required a direct contact of LPS with the respiratory epithelium [23]. We recently reported that systemic fetal inflammation can be induced by selective exposure of the fetal lung or the amniotic fluid to LPS [24]. However, the role of the isolated fetal GI tract in inducing systemic inflammation was not studied. This is an important question since the fetus swallows almost 50% of the amniotic fluid volume, exposing the GI tract epithelium (itself an important immunological organ) to large quantities of inflammatory products in the amniotic fluid [25].

Our objective was to quantify the contribution of the lung, gut and the chorioamnion/skin, in isolation, to the induction of systemic fetal inflammation caused by chorioamnionitis. We hypothesized that LPS exposure localized to different fetal organs will induce a differential fetal inflammatory response. Fetal sheep were instrumented with indwelling catheters to deliver either LPS or sterile saline to either the lung (isolated by tracheal occlusion), gastro-intestinal tract (isolated by oesophageal occlusion) or skin/amnion (isolated by occlusive snout seal). We analysed inflammatory markers in fetal plasma, liver, spleen and lung at 2 d or 6 d after LPS or saline exposure. The inflammatory responses were compared to intraamniotic LPS administration after sham fetal surgery.

## Materials and Methods

### Animals

All procedures involving animals were performed at The University of Western Australia (Perth, WA) following review and approval by the animal care and use committees of The University of Western Australia and Cincinnati Children’s Hospital (Cincinnati, OH.). Date mated Australian merino ewes with singleton pregnancies were randomised to fetal surgery and intervention groups (control or LPS) for either 2 d or 6 d. Ewes were delivered operatively at 124 d±2 d GA (approximately equivalent to 30 weeks human gestation), and euthanized with an intravenous injection of pentobarbitone (100 mg/kg). Fetal tissues for protein or mRNA expression analyses were rapidly dissected and snap frozen in liquid nitrogen for subsequent analysis. Fetal tissues for histological analysis were placed in embedding cassettes and fixed in 10% neutral buffered formalin for 24–48 hrs before being processed for paraffin embedding. Arterial cord blood pH, pO_2_ and pCO_2_ were measured on a Siemens Rapidlab1265 platform (Siemens, Munich, Germany).

### Surgical Procedures

Fetal recovery surgeries were performed using strict aseptic technique. Ewes were pre-medicated with an intra-muscular (IM) injection of buprenorphine (0.02 mg/kg) and acepromazine (0.01 mg/kg) for at least 30 minutes before induction of anaesthesia with an intravenous (IV) bolus of midazolam (0.25 mg/kg) and ketamine (5 mg/kg). Ewes were intubated and maintained on intermittent positive-pressure ventilation and anaesthesia using inhaled isofluorane. Heart rate, venous and arterial pressure (mmHg), end-tidal CO_2_ and SpO_2_ were constantly monitored and kept within normal physiological ranges. Maintenance intravenous fluids (normal saline) were infused at 10 mL/kg/h. A transdermal fentanyl patch (75 µg/h) provided post-operative analgesia, supplemented with morphine sulphate (0.1 mg/kg) as necessary. The experimental groups are summarised in Table 1. All anaesthetic/analgesic compounds used in this study were supplied by Provet WA (Perth, Western Australia).

**Table 1:** Summary of fetal surfaces exposed to intervention (either *E.coli* LPS or saline) in each surgical group.

		Experimental Group Characteristics		
Group Name	N	Surgical Procedure	Gestational Age (d) at Surgery	Fetal Organs Exposed to Intervention
2 d Gut	5	Gut isolation+stomach infusion	122	GI tract
6 d Gut	5	Gut isolation+stomach infusion	118	GI tract
2 d Lung	5	Lung isolation+tracheal infusion	122	Airways
6 d Lung	5	Lung isolation+tracheal infusion	118	Airways
2 d IA Ocln	7	Snout occlusion+intraamniotic infusion	122	Amniotic epithelium and skin
6 d IA Ocln	6	Snout occlusion+intraamniotic infusion	118	Amniotic epithelium and skin
2 d IA	5	Sham surgery+intraamniotic infusion	122	GI tract, airways, amniotic epithelium & skin
6 d IA	4	Sham surgery+intraamniotic infusion	118	GI tract, airways, amniotic epithelium & skin
Control	13	Combined*	118 or 122	GI tract, airways, amniotic epithelium & skin

* (Combined): represents pooled surgical controls from each intervention group, with fetal organ(s) exposed to sterile saline.

We dosed the fetus with LPS (*E.coli* O55:B5; Sigma-Aldrich, St. Loius, MO.) based on our previous experience and the physiology of fluid trafficking in the fetus. A dose of 10 mg LPS in the amniotic fluid consistently induces chorioamnionitis and lung inflammation/maturation [17], [26]. The lung epithelium will be exposed to amniotic fluid by the mixing of that fluid with fetal lung fluid during breathing, and we previously reported that 1 mg of LPS delivered by osmotic pump caused lung inflammation and maturation [23]. As the fetus swallows approximately 50% of total amniotic fluid volume every 24 h, we gave 5 mg LPS to the isolated fetal stomach [24].

#### Fetal gastro-intestinal tract isolation

The trachea was cannulated to drain fetal lung fluid to a bag as in lung surgery. The oesophagus was exposed and a catheter attached to a mini- osmotic pump (secured in a sub-dermal pocket) delivered intervention (5 mg LPS in 200 µL saline or 200 µL saline) to the fetal stomach over 24 h. The catheter was secured and the oesophagus superior to the catheter insertion site was ligated.

#### Fetal lung isolation

A 3 cm medial incision was made in raised skin overlying the cricoid cartilage. A ‘half-moon’ incision was made in a cartilage ring immediately inferior to the cricoid cartilage, allowing for the insertion of two occlusive catheters, one draining lung fluid into a collection bag sited in the amniotic cavity and a second attached to a mini- osmotic pump (secured in a sub-dermal pocket) delivering intervention (1 mg LPS in 200 µL saline or 200 µL saline) to the airways over 24 h. The trachea was occluded superior to the catheter site, catheters secured in place and incision sites were sutured.

#### Fetal snout occlusion

The trachea was cannulated to drain fetal lung fluid to a bag as in the lung surgery groups. A sterile, size 6 surgical glove (Ansell, Iselin, NJ.) was used to occlude the snout securely. A mini-osmotic pump was sutured to a hind limb to deliver intervention (10 mg LPS in 200 µL saline or 200 µL saline) to the amniotic fluid over 24 h.

#### Intra-amniotic

The surgical procedures were identical to those in the fetal snout occlusion group, however no seal was placed over the fetal snout. A mini-osmotic pump was sutured to a hind limb to deliver intervention (10 mg LPS in 200 µL saline or 200 µL saline) to the amniotic fluid over 24 h.

### Relative Quantification of mRNA Expression

Total RNA was isolated from fetal tissues homogenised in TRIzol (Life Technologies, Carlsbad, CA.) as previously reported [27]. mRNA transcripts were measured by quantitative PCR using single-stranded cDNA reverse transcribed from mRNA (Verso cDNA kit, Thermo Scientific, Waltham, MA.). Ovine specific PCR primers and hydrolysis probes for IL-1β, IL-6, IL-8, serum amalyoid protein A3 (SAA3), hepcidin, and C-reactive protein (Applied Biosystems, Carlsbad CA.) were used to perform quantitative PCR reactions (ABI Prism 7300, Applied Biosystems, Carlsbad CA.). Reaction cycling conditions were as follows: 2 min incubation at 50°C, followed by incubation at 95°C for 10 min (x 1), followed by 40 cycles of alternating temperatures of 95°C for 15 sec and 60°C for 1 min. Cq values were normalised to ribosomal 18 s RNA and expressed as fold changes relative to pooled control values. Reaction efficiencies were within limits proposed in the MIQE guidelines [28].

### Fetal Plasma Cytokine/Chemokine Measurement

Concentrations of fetal plasma cytokines were measured as previously described using sandwich enzyme-linked-immunosorbent assays (ELISA) employing the following antibodies: IL-1β (coating antibody, rabbit anti-ovine IL-1 β. Guinea pig anti-ovine IL-1β primary antibody [both Seven Hills Bioreagents, Cincinnati, OH.]); IL-6 (coating antibody, mouse anti-ovine IL-6 [MAB1004, Millipore, Billerica, MA.]. Rabbit anti-ovine IL-6 primary antibody [AB1839, Millipore, Billerica, MA]); IL-8 (coating antibody, mouse anti-ovine IL-8 [MAB10445, Millipore, Billerica, MA.]. Rabbit anti-ovine IL-8 primary antibody [Chemicon # AB1840]); monocyte chemotactant protein-1 (MCP-1) (rabbit anti-sheep MCP-1 coating antibody), guinea pig anti-sheep MCP-1 primary antibody (Seven Hills Bioreagents, Cincinnati, OH.) [29]. The detection antibody for all the assays was an appropriate specific HRP-conjugated antibody. The detection limits and the dynamic range of measurements were: IL-1ß - 0.20–12.0 ng/mL, IL-6 - 0.20–12.0 ng/mL, IL-8 - 0.40–25.0 ng/mL and MCP-1 0.1- 80 ng/mL). The correlation coefficient was 0.94–0.99 for all assays.

### Histology

5 µm-thick sections from formalin fixed tissues embedded in paraffin blocks were used for haemotoxylin and eosin staining with a standard serial protocol and final clearing in xylene. Sections for immunohistochemical analysis were deparaffinised and rehydrated before microwave-assisted antigen retrieval in citric acid buffer at pH 6.0. Endogenous peroxidase activity was blocked with CH_3_OH/H_2_O_2_ treatment. Sections were blocked with 2% goat serum in phosphate buffered saline (PBS). Sections were incubated for 16 h at 4?C with primary antibodies specific for either PU.1 (Sc-352, Santacruz Biotechnology CA, 1∶500 dilution), FOXP3 (14-7979-82, eBioscience, San Diego CA., 1∶50 dilution), CD3 (A0452, Dako, Glostrup, Denmark, 1∶100) or MPO (catalogue # CMC028 Cell Marque, Rocklin, CA., 1∶400) diluted in 2% goat serum in PBS. Sections were washed repeatedly in PBS before being incubated with an appropriate species specific secondary antibody (1∶200) for 30 minutes at room temperature. Slides were repeatedly washed in PBS before antigen:antibody complexes were visualized with a Vectastain ABC peroxidase Elite kit (Vector Laboratories Inc, Burlingame, CA.). Antigen detection was enhanced with nickel-diaminobenzidine, followed by incubation with TRIS-cobalt to give a black precipitate. Nuclei were counterstained with Nuclear Fast Red for photo-microscopy. Blind scoring of tissues was done by counting PU.1, FOXP3,CD-3 or MPO positive cells in 10 comparable non-overlapping high power fields for each animal.

### Haematology

Complete blood counts (CBC) and differential analyses were performed by VetPath Laboratory Services using an automated Coulter counter customised for sheep (Ascot, Perth, Western Australia).

### Statistical Analyses

All values are expressed as mean ± standard deviation. All analyses were performed using IBM SPSS Statistics for Windows, Version 20.0 (IBM Corp. Armonk, NY.). Continuous variable data were assessed for normality with Shapiro-Wilk tests. Parametric data were screened for outliers with Dixon’s Q-parameter and differences tested for significance with one-way ANOVA employing an *α*-value of 0.05. Non-parametric data were tested for significance with Kruskal-Wallis one-way ANOVA, with multiple comparisons performed using Rank-Sum tests and an *α*-value corrected for *n* multiple comparisons.

## Results

### Physiological Variables at Delivery

The maternal/fetal surgical mortality was 9% (7/75 animals). Fetal weights and cord arterial blood (CB) pH, pCO_2_ and pO_2_ values are presented in Table 2. No inter-group difference was observed for any variable. The abnormalities in pH and blood gas reflect euthanasia of the ewe prior to surgical delivery and resultant fetal respiratory acidosis.

**Table 2:** Physiological variables at delivery.

		O55:B5 *E.coli* LPS exposure							
	Control Saline	2d Gut	6d Gut	2d Lung	6d Lung	2d IA Ocln	6d IA Ocln	2d IA	6d IA
Fetal Weight, kg	2.9±0.4	2.8±0.4	3.1±0.5	2.80±0.3	2.8±0.4	3.0±0.3	3.10±0.3	3.0±0.1	2.70±0.3
CB pH	7.1±0.1	7.10±0.1	7.1±0.1	7.1±0.1	7.1±0.1	7.1±0.1	7.0±0.1	7.0±0.04	7.0±0.1
CB pCO_2_, mmHg	98.0±17.2	90.0±15.4	103.5±9.4	102.0±15.0	98.4±14.0	96.0±12.0	111.0±12.0	113.0±12.0	114. ±13.0
CB pO_2_, mmHg	4.5±3.2	2.9±1.0	1.8±1.0	3.0±2.0	3.0±3.0	6.3±3.0	4.4±3.3	3.0±2.0	4.0±3.0

CB: cord arterial blood.

### Fetal Lung Inflammation

**Figure 1:**
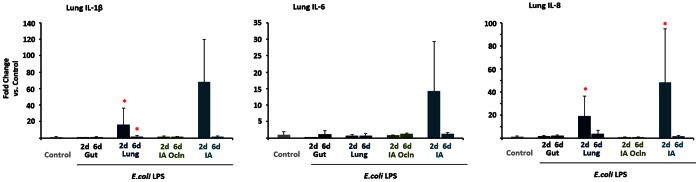
LPS infusion in the lung or the amniotic fluid induced lung cytokine expression. Quantification of messenger RNAs for IL-1β (A), IL-6 (B) and IL-8 (C) was performed by real-time PCR assays using sheep-specific primers and hydrolysis probes. Each cytokine was normalized to 18s ribosomal protein mRNA (internal control), and levels for each group were expressed relative to controls as fold increase relative to controls. Cytokine expression increased in the 2d “Lung” and the 2d “IA” LPS groups (*p<0.0125 vs. control for IL-1β and IL-8, *p*<0.05 for IL-6 vs. control).

Lung inflammation was evaluated by measuring expression of select pro-inflammatory cytokines and by histological evaluation for activated inflammatory cells. Increases in IL-1β (20–40 fold) and IL-8 mRNA (20–50 fold) were identified in the ‘Lung’ and ‘IA’ LPS exposure groups only (Figure 1). IL-6 mRNA was not different between the groups exposed to LPS (there was a variable non-significant increase in IL-6 in the ‘IA’ LPS group). Consistent with the mRNA data, increased levels of IL-1β, and IL-8 protein in the bronchoalveolar lavage fluid (BALF) were detected only in the ‘Lung’ and ‘IA’ LPS exposure groups (Table 3). Bronchoalveolar lavage fluid MCP-1 also increased only in the ‘lung’ and ‘IA’ LPS groups. Both the RNA and protein measurements were higher at 2 d compared to 6 d following LPS.

**Table 3:** Fetal lung bronchoalveolar lavage cytokine/chemokine levels (ELISA).

		O55:B5 *E.coli* LPS exposure							
	Control Saline	2d Gut	6d Gut	2d Lung	6d Lung	2d IA Ocln	6d IA Ocln	2d IA	6d IA
BALF IL-1β, ng/ml	0.8±1.7	0.4±0.4	6.7±14	**30.5±23.7** *	**17.4±18.6** *	0.0±0.0	0.0±0.0	**37.4±32.3** *	**9.3±7.4** *
BALF IL-6, ng/ml	0.2±0.2	0.4±0.2	0.3±0.2	0.2±0.2	0.2±0.1	0.0±0.0	0.03±0.02	0.1±0.1	0.06±0.04
BALF IL-8, ng/ml	5.7±7.3	1.0±1.3	3.9±7.9	**16.4±2.0** *	**16.1±1.9** *	**0.0±0.0** *	**0.01±.02** *	**22.9±0.8** *	**20.0±2.4** *
BALF MCP-1 ng/ml	0.8±1.7	0.4±0.4	6.7±14.3	**30.5±23.7** *	**17.4±18.6** *	0.0±0.0	0.0±0.0	**37.4±32.3** *	**9.3±7.5** *

BALF: bronchoalveolar lavage.

* *p*<0.0125 vs. control.

**Figure 2:**
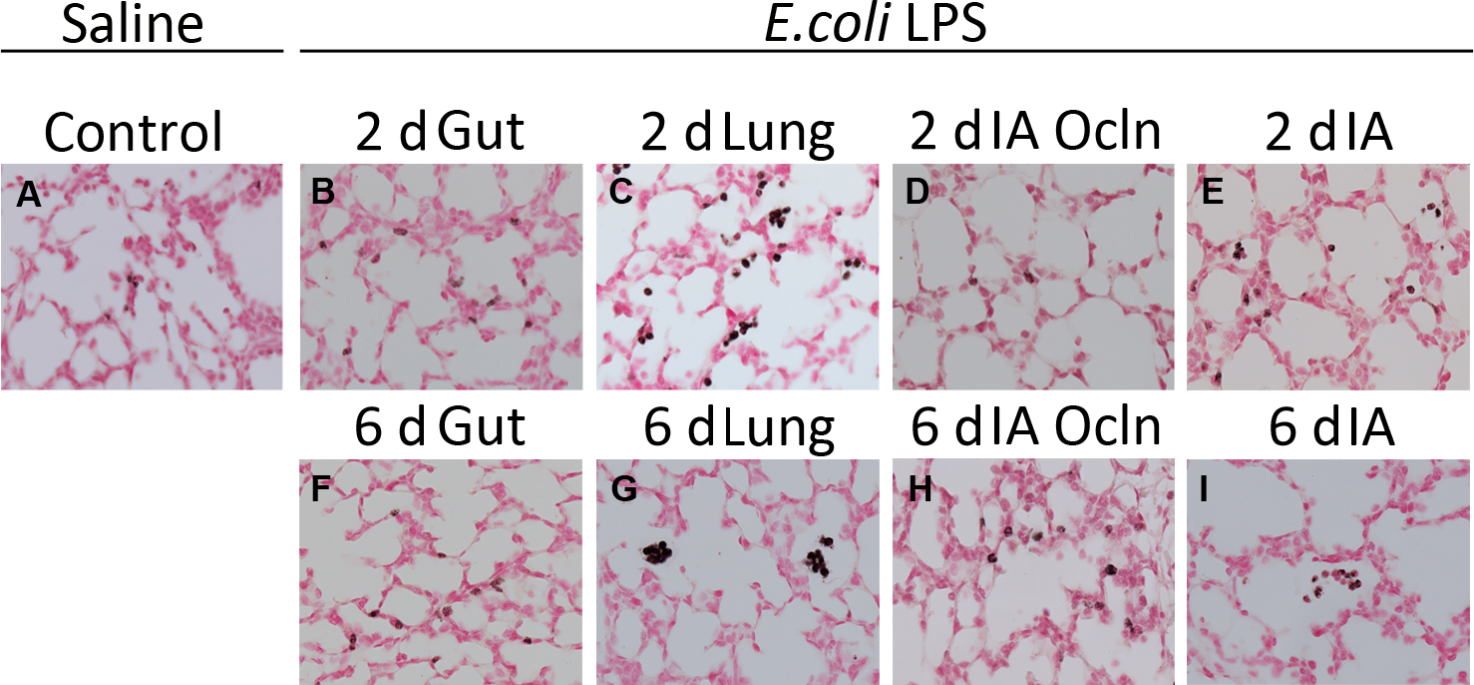
LPS infusion in the lung or the amniotic fluid induced myeloperoxidase (MPO) expression in the lung. Representative photomicrographs are shown for MPO immunostaining in saline control exposed lung (A), 2d (B) and 6d (F) “Gut”, 2d (C) and 6d (G) “Lung”, 2d (D) and 6d (H) “IA Ocln” and 2d (E) and 6d (I) “IA” groups. All images 20×magnification. The immunostained inflammatory cells (exhibiting dark brown staining) were increased in the 2d and 6d “Lung”, 2d “IA” LPS and 2d “Gut” group, relative to controls (see quantitative counts of MPO positive cells in all the groups in Table 4).

To better understand the cellular inflammatory response and activation in the lung, inflammatory cell counts were performed for cells expressing CD3+ (T-cells), PU.1 (a maturation marker for monocytes), and myeloperoxidase (MPO) (a marker for activated neutrophils and monocytes) (Table 4). CD3+ T-cell infiltration was only detected in the ‘Lung’ LPS group. PU.1 positive cell counts were increased in both the ‘Lung’ and ‘IA’ LPS groups. No changes in proportion of cells staining for FOXP3 were identified in any of the exposure groups relative to control. Relative to pooled control, counts for MPO-positive cells were significantly (*p*<0.05) increased at 2 d and 6 d in the ‘IA’ and ‘Lung’ LPS groups and at 2d in the ‘Gut’ LPS group. Representative images for anti-MPO stained preparations from 6 d groups are presented in Figure 2.

**Table 4:** Fetal lung immunocyte counts.

		O55:B5 *E.coli* LPS exposure							
	Control Saline	2d Gut	6d Gut	2d Lung	6d Lung	2d IA Ocln	6d IA Ocln	2d IA	6d IA
Lung MPO+, HPV	4.3±1.7	**14.3±5.2** *	14.2±4.6	**49.0±8.2** *	**30.3±9.8** *	2.7±1.7	14.6±8.4	**18.0±6.4** *	9.2±4.3
Lung CD3+, HPV	8.8±5.5	14.5±5.2	31.0±37.0	**39.0±10.0** *	**24.0±9.0** *	3.0±1.8	4.6±1.4	13.6±5.1	9.6±5.3
Lung PU.1, HPV	21.0±10.0	27.3±11.6	90.6±123.1	**66.2±10.5** *	**37.0±9.5** *	8.00±2.2	28.4±1.9	**65.4±40.9** *	31.0±10.0

HPV: cells per high powered view.

* *p*<0.05 vs. control.

### Inflammatory Markers in the Blood

**Figure 3:**
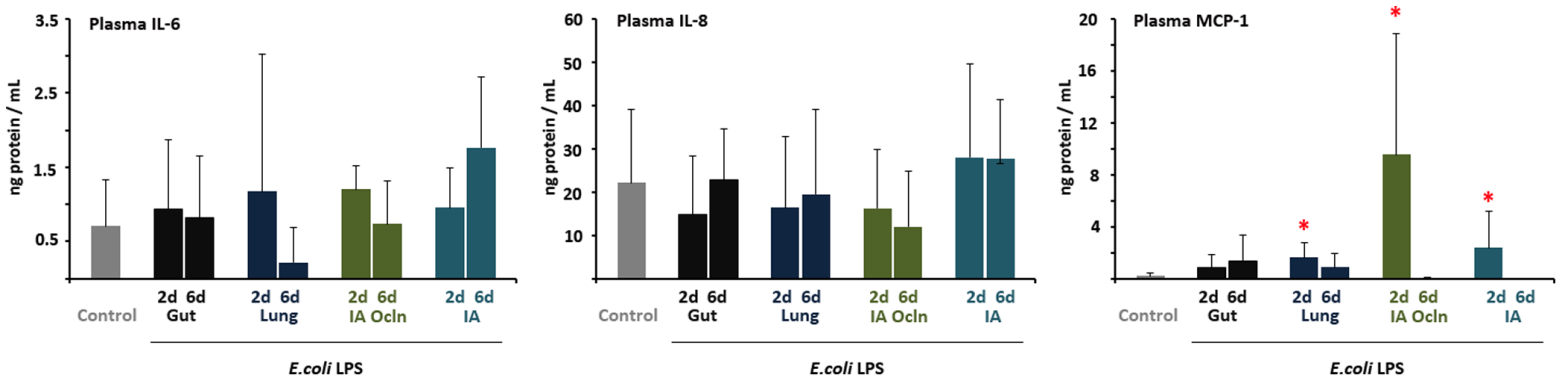
LPS infusion in the lung or the amniotic fluid increased MCP-1 levels in the plasma. IL-6, IL-8 and MCP-1 protein levels in the plasma were measured by ELISA, (A) IL-6 and (B) IL-8 and (C) MCP-1. MCP-1 but not IL-6 or IL-8 levels increased in the plasma in the 2d “Lung”, 2d “IA Ocln” and the 2d “IA” LPS groups (*p<0.0125 vs. control for MCP-1, *p*<0.05 vs. control for IL-6 and IL-8).

Compared to controls, leucocytes were increased in the 6 d ‘Lung’ (*p* = 0.003) and ‘IA Ocln’ animals (*p* = 0.004). Consistent with migration to tissues, the neutrophil counts decreased at 2 d in the ‘IA Ocln’ group (*p* = 0.001). Consistent with neutrophilia at 7 d after IA LPS reported previously [17], neutrophil counts were significantly increased in 6 d ‘Lung’ animals (*p* = 0.007). Relative to control, monocyte counts were also increased in 6 d ‘Lung’ animals (*p* = 0.007) and approached significance in 6 d ‘IA Ocln’ animals (*p* = 0.016; *α = 0.012*) (Table 5). Plasma cytokines were measured as an indication of systemic inflammation. Relative to controls, 2 d LPS exposure resulted in significantly increased concentrations of plasma MCP-1 in ‘Lung’, ‘IA Ocln’ and ‘IA’ groups (*p* = 0.000, 0.000 and 0.007, respectively). However, LPS exposure did not elicit change in the plasma concentrations of either IL-6 or IL-8 in any of the experimental groups (Figure 3).

**Table 5:** Total and differential white blood cell counts.

		O55:B5 *E.coli* LPS exposure							
	Control Saline	2d Gut	6d Gut	2d Lung	6d Lung	2d IA Ocln	6d IA Ocln	2d IA	6d IA
Total WBC, 10^9^/l	4.3±3.0	6.0±2.0	6. ±2.5	4.0±1.5	**10.0±7.0** *	2.9±1.0	**9.0±3.9** *	2.2±0.1	5.0±1.9
Neutrophils, 10^9^/l	1.7±2.5	2.5±2.1	4.0±2.4	0.9±0.7	**7.0±7.3** *	**0.1±0.01** *	3.5±2.6	0.2±0.1	2.30±1.0
Lymphocytes, 10^9^/l	2.0±1.0	2.3±0.6	1.6±0.4	2.1±1.0	2.4±1.2	1.8±0.4	3.2±1.3	1.7±0.8	1.80±0.7
Monocytes, 10^9^/l	0.2±0.3	0.3±0.2	0.4±0.4	0.2±0.2	**0.6±0.6** *	0.1±0.03	**1.0±0.60** #	0.1±0.1	0.2±0.06

Cell counts (10^9^/L) from fetal arterial cord blood taken at delivery.

* *p*<0.0125 vs. control;

# approached significance (*p* = 0.016).

### Inflammation in the Fetal Spleen

**Figure 4:**
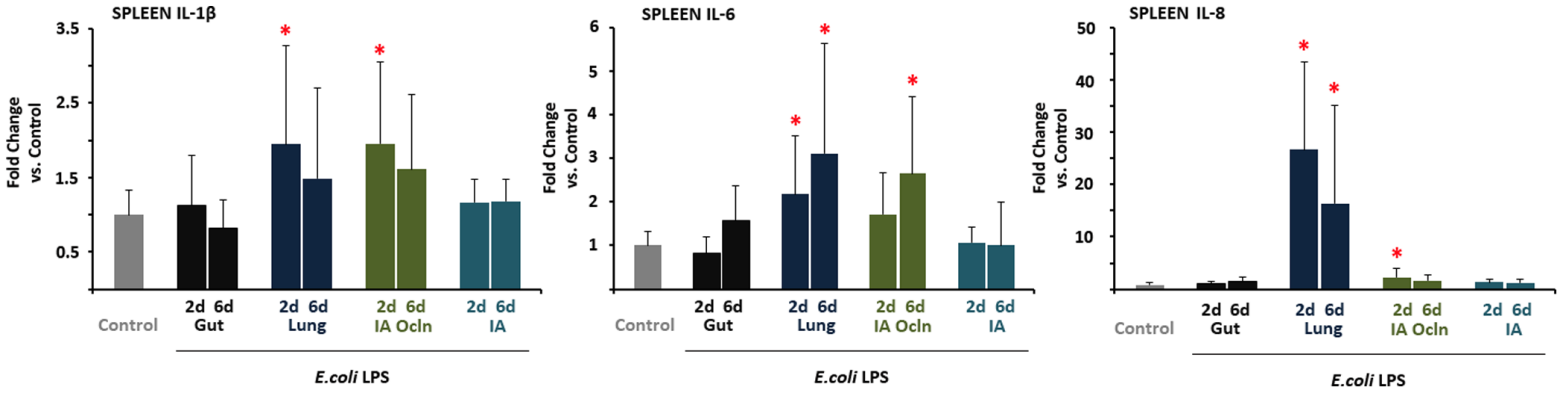
LPS infusion in the lung or the amniotic fluid induced spleen cytokine expression. Quantification of messenger RNAs for (A) IL-1β, (B) IL-6, (C) IL-8, was performed by real-time PCR assays using sheep-specific primers and hydrolysis probes. Each cytokine was normalized to 18s ribosomal protein mRNA (internal control), and levels for each group were expressed relative to controls as fold increase relative to controls. Cytokine expression increased in the “Lung” and the “IA Ocln” LPS groups. (*p<0.0125 vs. control for IL-6 and IL-8, *p*<0.05 vs. control for IL-1β).

In the fetal spleen, relative to controls, the 2 d LPS exposure resulted in significantly increased IL-1β and IL-8 mRNAs in ‘Lung’ and ‘IA Ocln’ groups, while IL-6 mRNAs were induced in the ‘Lung’ group only (Figure 4). The 6d LPS exposure resulted in significantly increased IL-6 and IL-8 mRNAs in the ‘Lung’ group, while IL-6 mRNA also increased in the 6 d ‘IA Ocln’ group. No significant changes in cytokine mRNA expression were identified in ‘Gut’ and ‘IA’ LPS animals.

### Inflammation in the Fetal Liver

**Figure 5:**
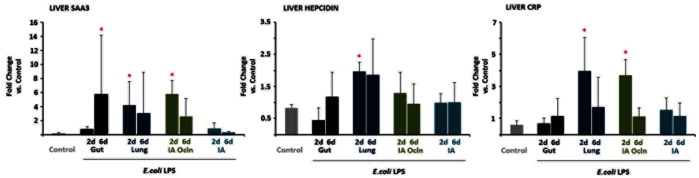
LPS infusion in the gut or the lung or the amniotic fluid induced liver acute phase reactant gene expression. Quantification of messenger RNAs for (*A*) serum amyloid A3 (SAA3) (*B*) hepcidin and (*C*) c-reactive protein (CRP) was performed by real-time PCR assays using sheep-specific primers and hydrolysis probes. Each cytokine was normalized to 18s ribosomal protein mRNA (internal control), and levels for each group were expressed relative to controls as fold increase relative to controls. Liver acute phase response was detected in the 6d “Gut”, 2d “Lung” and the 2d “IA Ocln” LPS groups (*p<0.05 versus controls).

Induction of hepatic inflammation was assessed by measuring mRNA expression of acute phase proteins (Figure 5). Relative to controls, at 2 d after LPS exposure, serum amyloid A3, and C-reactive protein mRNA increased in the ‘Lung’ and ‘IA Ocln’ groups, while hepcidin mRNA increased only in the ‘Lung’ group. Interestingly, the only acute phase reactant response at 6d after LPS exposure was an increase in serum amyloid A3 mRNA in the ‘Gut’ group.

## Discussion

Although chorioamnionitis is diagnosed in more than 50% of women who deliver prior to 30 weeks of gestation [5], the incidence of early onset sepsis in preterm infants <30 weeks gestation is about 2% [30]. The responses in sheep to intraamniotic injection of LPS are quite different from intravascular injection: Intraamniotic injection of 100 mg LPS is tolerated without any overt clinical deterioration, while a 10,000 fold lower dose is fatal if delivered intravenously [26]. Taken together, these data strongly that the vascular system is much more responsive to LPS compared to the fetal tissues exposed to the amniotic fluid. An important, unresolved issue regarding perinatal infections is thus which fetal tissues are important in transducing inflammatory responses to organs not in direct communication with the amniotic fluid e.g. the brain or the blood.The lung, gastro-intestinal tract, the amniotic membrane and skin are the four major fetal surfaces exposed to the amniotic fluid. A key finding from our study is that LPS-driven inflammation of the fetal lung (‘Lung’ group) and fetal skin/amniotic fluid (‘IA Ocln’ group), but not the fetal gastro-intestinal tract (‘Gut’ group), elicits an acute multi-organ systemic fetal inflammatory response. Our experiment is unique in using a fetal surgical approach to understanding the contributions of individual fetal organs to systemic fetal inflammation in chorioamnionitis.

The lung is an important organ in fetal inflammatory response in experimental models of chorioamnionitis. Following exposure to intraamniotic LPS, fetal sheep have lung inflammation [17], which results in lung maturation characterised by increases in airway surfactant and improved mechanical properties of the lung [17], [31]. However, the lung maturation is also associated with the expression of developmental genes that impair lung development, resulting in a decrease in the number of alveolar units [18], [32]. Lung inflammation and maturation can also be induced by intraamniotic injection of IL-1 [33]. We have reported previously that lung monocytes functionally mature to macrophages after exposure to intraamniotic LPS [34]. Together, these results suggest that multiple responses occur in the fetal lung following direct exposure to inflammatory stimuli during chorioamnionitis.

Both lung inflammation and lung maturation require contact of the inflammatory mediator with the airway epithelium, as surgical isolation of the lung, combined with intraamniotic LPS did not cause lung inflammation or maturation [23], [24]. Consistent with these results, in the present study, large increases in lung IL-1β and IL-8 mRNA and BALF IL-1β, IL-8 and MCP-1 were identified only in the ‘Lung’ and ‘IA’ LPS exposure groups, which allowed direct exposure of LPS to fetal airway epithelium. Further, we previously reported that inhibition of IL-1 signalling largely inhibited intraamniotic LPS induced lung inflammation, maturation and systemic inflammation. Our present data demonstrate that selective infusion of LPS in the fetal lung is sufficient to induce a multi-organ fetal systemic inflammation. Taken together, these experiments demonstrate that IL-1 signalling in the lung is an important mechanism in the transduction of fetal inflammation following intraamniotic LPS injection.

The gastrointestinal tract has a large surface area and is exposed to the contents of the amniotic fluid since fetuses swallow approximately 50% of the amniotic fluid in 24 hours [25]. Mucosal inflammatory responses to microbial agonist are, by virtue of the gastro-intestinal tract’s constant exposure to microorganisms, tightly regulated [35]. This dampening control may be reflected in the apparent hyporesponsiveness of the preterm fetal ovine gastro-intestinal tract to acute LPS exposure in the present study. Wolfs *et al*. reported intestinal inflammation with activated neutrophils, CD3+ T-cell and gamma-delta T-cell infiltration, decreased immunosuppressive T-regulatory cells, and disrupted tight junctions after exposure to intraamniotic LPS or IL-1 [20]. Interestingly, these intestinal inflammatory responses were not apparent at 2 d, but occurred 3–14 d after exposures. Davies *et al*., using an endocervical rabbit model of acute LPS-driven intra-amniotic infection, reported an absence of histological gastro-intestinal inflammation in fetuses autopsied between 0 and 30 hours post-innoculation [36]. These results demonstrate a time-dependent response of the fetal gut to intraamniotic inflammatory stimuli. We did not detect systemic inflammatory responses after selective exposure of the GI tract to LPS either at 2 d or 6 d after exposures (with a sole exception of increased serum amyloid A3 expression in the liver 6 d after exposure). It is, however, possible that subtle responses could have been missed since we measured selected indicators of systemic inflammation. Regardless, the conclusion from our data, taken together with previous reports is that the fetal GI tract responds to chorioamnionitis with modest localized inflammation. In the present study, the fetal GI tract did not make a significant contribution to the transduction of systemic inflammation, relative to that resulting from LPS exposure of the fetal lung and skin/chorioamnion.

Since our focus was on acute inflammation, the main indicators of fetal inflammation were the expression of pro-inflammatory cytokines in multiple fetal organs, acute phase reactant proteins in the liver, and changes in circulating leukocytes. CRP and SAA3 are acute phase proteins expressed in the liver and extra-hepatic tissues in response to inflammation. Both proteins, along with hepcidin (a multifunctional anti-microbial peptide) are up-regulated by inflammation and LPS-TLR-4 signalling [37], [38]. These acute phase reactants were significantly up-regulated in 2 d ‘Lung’ and ‘IA Ocln’ LPS-exposure groups. Splenic involvement characterised by morphological abnormalities, leukocyte depletion and aberrant splenic vein flow has been identified in human preterm labour and delivery in association with chorioamnionitis, funisitis and sepsis [39], [40]. We identified a splenic response to selective LPS exposure in the ‘Lung’ and ‘IA Ocln’ groups by increases in spleen IL-1β, IL-6 and IL-8 mRNA expression. We previously reported that chorioamnionitis induces a time-dependent increase in the ability of preterm fetal lung and blood monocytes to respond to LPS and TNFα [31], [41]. Intraamniotic LPS also decreased the thymic cortico-medullary ratio, activated thymic T-cells and decreased thymic T-regulatory immunosuppressive cells [42]. Thus LPS-induced chorioamnionitis can induce immune modulation. Whether these immunomodulatory changes are dependent on specific fetal organ inflammation e.g. lung vs. gut vs. chorioamnion is not known.

Both the amnion and skin epithelia of the fetus are in intimate contact with the amniotic fluid. We previously reported that both the skin and the chorioamnion respond to intraamniotic LPS with recruitment of inflammatory cells, and increased expression of pro-inflammatory cytokines [21], [43]. In this study, the experimental ‘IA’ and ‘IA Ocln’ experimental groups allowed us to assess the contribution of the chorioamnion and skin to systemic fetal inflammation (we did not isolate the fetal skin from the amnion). Interestingly, lung cytokine mRNAs were not induced in the ‘IA Ocln’ group. These experiments demonstrate that the chorioamnion and/or skin inflammation can contribute to systemic but not pulmonary inflammation in chorioamnionitis. The ‘IA’ group represents exposure of the amniotic fluid (and thus LPS) to the amnion, skin, lung and GI tract. Curiously, unlike the ‘Lung’ group, little systemic inflammation was identified in the ‘IA’ group, aside from increasing plasma MCP-1 levels at 2 d. However, similar to the ‘Lung’ group, large increases in lung IL-1ß, IL-6 and IL-8 mRNAs were detected in the ‘IA’ LPS group, demonstrating that the fetal lung is an effective transducer of systemic inflammation.

The systemic inflammation detected in the ‘IA’ LPS group is qualitatively and quantitatively lower than that demonstrated in our previous studies with a bolus injection of 10 mg intraamniotic LPS in non-surgical animals [44]. We hypothesise that the overall lack of systemic response in the ‘IA’ group is likely due to different responses to a bolus LPS infusion in non-surgical animals vs. a slow 24 h infusion in the surgical animals. Although surgery and anaesthesia could be confounding factors, the systemic inflammatory responses in ‘Lung’ and ‘IA Ocln’ surgical animals argue against this possibility. It is important to note that these studies employed LPS as a sterile pro-inflammatory agent. Although this allowed us to deliver a standardised inflammatory insult, this system likely deviates somewhat from the inflammatory response that would be generated by a viable, replicating and potentially invasive microorganism. Regardless, these data demonstrate that the chorioamnion and skin are capable of contributing to systemic inflammation in the fetus exposed to chorioamnionitis. A note-worthy aspect of our study is that all the groups had a similar fetal surgery with the same anaesthesia and post-operative management. This is important because, surgical intervention in pregnant sheep has been reported to impact the fetal birth-weight and pregnancy development [45].

In addition to extending an understanding of how FIRS might be propagated, these findings are of importance to on-going efforts to develop interventions for preterm birth. Since the organs in contact with amniotic fluid induce somewhat different inflammatory response, these contributions to fetal injury responses and subsequent pathologies likely require further investigation. As a general strategy, delivery of immunomodulators and anti-inflammatory therapies in the amniotic fluid might be an attractive strategy to reduce chorioamnionitis associated morbidity.
